# Case Report: Kidney preservation using immune checkpoint inhibitors in Muir–Torré syndrome-associated upper tract urothelial carcinoma, with literature review

**DOI:** 10.3389/fimmu.2026.1784811

**Published:** 2026-06-18

**Authors:** Majd Hanna, Gil Bar-Sela

**Affiliations:** 1 Cancer Center, Emek Medical Center, Afula, Israel; 2 Bruce Rappaport Faculty of Medicine, Technion-Israel Institute of Technology, Haifa, Israel

**Keywords:** case report, immunotherapy, mismatch repair deficiency, Muir–Torré syndrome, non-rectal cancer, organ preservation, pembrolizumab, upper tract urothelial carcinoma

## Abstract

Mismatch repair–deficient tumors exhibit high sensitivity to immune checkpoint inhibitors. While “watch and wait” organ preservation strategies following clinical complete response are increasingly established in rectal cancer, their application to non-rectal malignancies remains experimental. This report summarizes emerging evidence regarding non-operative management, specifically recent pivotal data on tumor-agnostic responses, and contextualizes these findings through a representative case of MSH6-deficient upper tract urothelial carcinoma. Recent prospective data suggest early substantial clinical complete response rates in selected non-rectal mismatch repair–deficient tumors (approximately 65%), enabling initial non-operative management in selected patients. We present a case of high-grade upper tract urothelial carcinoma in a patient with Muir–Torré syndrome treated with definitive pembrolizumab. The patient achieved a deep clinical response characterized by complete metabolic and endoscopic resolution, with no evidence of invasive carcinoma on biopsy, resulting in successful kidney preservation without surgery. Immunotherapy-based organ preservation may represent a viable strategy for highly selected patients with mismatch repair deficiency. This approach offers a potential selective alternative to immediate radical nephroureterectomy in deep responders, provided that strict and intensive surveillance protocols are feasible.

## 1 Introduction

Muir–Torré syndrome (MTS) is a rare autosomal dominant variant of Lynch syndrome characterized by the coexistence of sebaceous skin neoplasms and internal malignancies (1). It results from germline defects in DNA mismatch repair (MMR) genes, most commonly *MSH2*, *MLH1*, and *MSH6* (2). Upper tract urothelial carcinoma (UTUC) is a recognized component of the Lynch syndrome spectrum, particularly in *MSH2* and *MSH6* carriers (3). The *MSH6* genotype is of particular relevance to organ-sparing discussions; carriers often exhibit an attenuated risk of colorectal cancer compared to *MLH1*/*MSH2* carriers but maintain a persistent risk for extracolonic malignancies, specifically UTUC (3).

Radical nephroureterectomy (RNU) with bladder cuff excision remains the standard surgical approach for high-grade UTUC (4). However, RNU renders patients functionally mononephric, increasing the risk of chronic kidney disease and cardiovascular morbidity, which may preclude future systemic therapies (5). Consequently, there is a critical need for organ-sparing alternatives. Historically, the “watch and wait” strategy pioneered by Habr-Gama et al. (2004) for rectal cancer demonstrated that clinical complete response (cCR) could translate into organ preservation (6).

In the era of precision medicine, immune checkpoint inhibitors (ICIs) have become the cornerstone of treatment for dMMR malignancies due to their high neoantigen load (7). The efficacy of pembrolizumab in non-colorectal dMMR cancers was firmly established in the Keynote-158 study (8). More recently, Cercek et al. (2022) reported a 100% cCR rate in dMMR rectal cancer with neoadjuvant PD-1 blockade, allowing patients to avoid surgery entirely (9). Emerging prospective data have begun to expand this “rectal model” to a tumor-agnostic “non-operative” paradigm (10, 11). This report presents a unique case of MSH6-deficient UTUC successfully managed with definitive immunotherapy, contributing to the evolving landscape of organ preservation.

## 2 Case description

A 55-year-old man with a medical history of type 2 diabetes mellitus and Muir–Torré syndrome presented for evaluation of a right ureteral mass. The diagnosis of MTS was established based on a 20-year history of recurrent sebaceous neoplasms, including two aggressive sebaceous carcinomas excised in 2017 and 2022. Immunohistochemistry (IHC) of the skin lesions consistently demonstrated a loss of MSH6 protein expression. The patient was under regular oncologic surveillance. The lesion was detected during surveillance imaging; the patient denied hematuria, flank pain, or constitutional symptoms. Physical examination was unremarkable, with no palpable abdominal masses, costovertebral angle tenderness, lymphadenopathy, or other clinically significant findings. In 2021, a comprehensive evaluation including colonoscopy, gastroscopy, abdominal ultrasound, and serum tumor markers was unremarkable. The clinical course is summarized in **Table 1**.

**Table 1:** Timeline of the episode of care.

Date	Event	Outcome/findings
2017–2022	Medical history	Diagnosis of Muir–Torré syndrome; excision of two MSH6-deficient sebaceous carcinomas
2021	Surveillance	Colonoscopy, gastroscopy, and ultrasound unremarkable
Feb 2025	Imaging	CT urography revealed a ureteral mass
Feb 2025	Diagnosis	Ureteroscopy and biopsy confirmed high-grade urothelial carcinoma with loss of MSH6
Mar 2025	Surgical consultation	Radical nephroureterectomy recommended; patient declined
Sep 2025	Treatment initiation	Pembrolizumab started (200 mg IV every 3 weeks)
Dec 2025	Re-evaluation	PET-CT: complete metabolic response; ureteroscopy: normal mucosa; cytology negative; biopsy: atypia without invasive carcinoma
Dec 2025	Follow-up	Disease-free with preserved renal function; ongoing immunotherapy
30 Apr 2026	Follow-up PET-CT	Complete metabolic remission maintained; no abnormal FDG uptake

## 3 Diagnostic assessment

In February 2025, computed tomography (CT) urography revealed a soft tissue mass in the middle third of the right ureter. Ureteroscopy with biopsy confirmed high-grade urothelial carcinoma. Staging PET-CT demonstrated localized hypermetabolic activity in the right ureter and renal pelvis, with no evidence of distant metastasis. IHC of the ureteral tumor confirmed loss of MSH6 expression, consistent with the patient’s germline deficiency. The diagnosis was established through concordant radiologic, endoscopic, histopathologic, and immunohistochemical findings. Pathologic assessment was performed by a board-certified pathologist.

## 4 Therapeutic intervention outcomes

Although radical nephroureterectomy (RNU) was recommended as standard treatment, the case was discussed at a multidisciplinary tumor board including specialists in urology, medical oncology and radiology. Following multidisciplinary discussion and patient counseling, the patient declined surgery and elected to pursue an immunotherapy-based organ-preservation strategy. Given the dMMR status and emerging data supporting organ preservation, he was treated with pembrolizumab (200 mg intravenously every 3 weeks) starting September 2025. After five cycles, re-evaluation in December 2025 demonstrated a deep clinical response with no evidence of invasive disease.

Radiological assessment via PET-CT showed complete metabolic resolution. Endoscopic evaluation via ureteroscopy revealed normal mucosa, and urine cytology was negative for malignant cells. Pathology from endoscopic biopsies demonstrated atypical urothelial proliferation with nested growth, suspicious for high-grade dysplasia, but no definitive carcinoma and no evidence of invasive disease. At the time of manuscript revision, the patient remained on pembrolizumab therapy and continued to demonstrate no evidence of disease progression. Follow-up PET-CT performed on 30 April 2026 demonstrated sustained complete metabolic remission with no abnormal FDG uptake. Pembrolizumab was well tolerated, with no immune-related adverse events, treatment interruptions, or dose modifications. Renal function remained preserved during treatment, with serum creatinine measuring 1.25 mg/dL (eGFR 60 mL/min/1.73 m²) prior to treatment initiation and 1.20 mg/dL (eGFR 63 mL/min/1.73 m²) at the December 2025 reassessment. The patient remained adherent to treatment and surveillance recommendations. Discussion.

This case illustrates the successful application of a non-operative strategy to the upper urinary tract, conceptually analogous to the paradigm shift observed in rectal cancer. Our patient’s outcome aligns with the newly published data by Cercek et al. (2025), which reported high rates of cCR in non-rectal tumors treated with PD-1 blockade. In that cohort, no patients progressed to unresectable disease while receiving immunotherapy, supporting a monitored non-operative approach in selected cases (11). In the present case, deep clinical response was pragmatically defined by complete metabolic resolution on PET-CT, normal ureteroscopic appearance, negative urine cytology, and absence of invasive carcinoma on repeat biopsy. We acknowledge that residual atypical urothelial changes do not meet strict pathological complete response criteria and therefore require continued surveillance.

### 4.1 Review of emerging clinical evidence

To contextualize this case, we reviewed pivotal prospective data and high-impact reports from 2004–2025 regarding immunotherapy-driven organ preservation. The feasibility of non-rectal organ preservation was recently supported by early results of a prospective Phase II study by Cercek et al. (2025), which evaluated neoadjuvant dostarlimab in dMMR solid tumor (11). Tumor-agnostic efficacy was observed in the non-rectal cohort, in which approximately 65% (35/54) of patients achieved a clinical complete response. Among complete responders, non-operative management was frequently pursued; 33 of the 35 patients elected to avoid surgical resection of organs such as the stomach and colon (11). Notably, the urothelial carcinoma subgroup (n = 7) demonstrated a 100% clinical complete response rate, providing early support for kidney-sparing approaches in this population. Key studies informing this paradigm shift are summarized in **Table 2**.

**Table 2:** Summary of selected pivotal studies informing the present case.

Study	Population	Tumor site	Immune checkpoint inhibitor	Key findings
Cercek et al. (2022) (9)	Locally advanced cancer (n = 12)	Rectum	Dostarlimab	100% clinical complete response (cCR) rate. Established the watch-and-wait paradigm for dMMR rectal cancer.
Cercek et al. (2025) (11)	Pan-tumor cohort (n =117)	Rectal and non-rectal	Dostarlimab	Non-rectal cohort achieved an approximate 65% cCR rate. The urothelial subgroup (n = 7) demonstrated a 100% cCR rate, supporting tumor-agnostic organ preservation.
Ikarashi et al. (2020) (12)	Case report	Upper tract urothelial	Pembrolizumab	Neoadjuvant therapy resulted in pathological complete response (pT0); however, radical nephroureterectomy was still performed, demonstrating drug efficacy without organ preservation.
Chalabi et al. (2020) (13)	Early-stage colon cancer (dMMR cohort, n = 20)	Colon	Nivolumab plus ipilimumab	100% pathological response (20/20) and 95% major pathological response (MPR) in dMMR tumors; treatment was feasible with no delays to planned surgery.
Marabelle et al. (2020) (8)	Advanced dMMR (n = 233)	Multiple non-rectal sites	Pembrolizumab	Durable objective responses across 27 tumor types (KEYNOTE-158), validating PD-1 blockade as a tissue-agnostic therapy.
Our case (2025)	Case report	Upper tract urothelial	Pembrolizumab	Deep clinical, radiologic, and endoscopic response with no evidence of invasive disease. Sustained complete metabolic remission was confirmed on follow-up PET-CT performed on 30 April 2026, allowing avoidance of radical nephroureterectomy and preservation of renal function.

The studies listed represent varying levels of evidence, from case reports to prospective trials, and include both neoadjuvant and definitive treatment settings.

### 4.2 Biological rationale and emerging evidence

The potential for organ preservation in dMMR tumors is driven by biology rather than anatomy. As described by Tanegashima et al. (2025), resectable dMMR/MSI-H cancers possess a unique, highly immunogenic tumor microenvironment (10). This environment allows for deep pathological responses to PD-1 blockade, often leaving no residual viable tumor cells and suggesting that radical surgery may not be mandatory for all complete responders. The prospective Phase II study by Cercek et al. (2025) supports this, demonstrating a tumor-agnostic efficacy where approximately 65% (35/54) of non-rectal patients achieved a clinical complete response. Notably, the urothelial carcinoma subgroup (n = 7) in their study demonstrated a 100% clinical complete response rate, providing early support for kidney-sparing approaches in this population (11).

### 4.3 Distinct features of this case

While our findings support the general conclusions of recent trials, this case offers distinct insights. Unlike the broad dMMR population in many trials, our patient carries a germline *MSH6* mutation. This genotype is increasingly recognized for its preferential association with urothelial rather than colorectal malignancies (3). Therefore, the development of tailored organ-preservation protocols is of disproportionate importance to this specific genetic subgroup. Whether responses differ among individual mismatch repair genes remains uncertain. Most available organ-preservation data combine tumors harboring MSH2, MLH1, MSH6, and PMS2 alterations. Although MSH6-associated urothelial malignancies may exhibit distinct clinical characteristics, current evidence is insufficient to determine whether response rates to immune checkpoint inhibition differ according to the specific mismatch repair gene involved. Furthermore, our case differs from the report by Ikarashi et al., in which pembrolizumab induced a pathological complete response but radical nephroureterectomy was ultimately performed. In contrast, our patient declined surgery and remained under active surveillance, providing a rare example of actual kidney preservation following immunotherapy rather than demonstration of treatment efficacy alone.

### 4.4 Surveillance challenges in the upper tract

A major challenge in translating the “rectal model” to UTUC is the difficulty of monitoring. In the established “watch and wait” protocol for rectal cancer, strict surveillance relies on the high accessibility of the rectum, allowing for digital rectal examination and direct endoscopy every 3–4 months (6, 9). Unlike the rectum, the upper tract is notoriously difficult to survey. Consequently, implementing non-operative management requires a rigorous and standardized surveillance framework to ensure patient safety. In the absence of established guidelines, we propose that intensive surveillance is mandatory: this should likely include a combination of cross-sectional imaging (CT Urography), serial PET-CT (where available and clinically justified), ureteroscopy, and selective urine cytology at frequent intervals (e.g., every 3–6 months) during the first two years. Any radiologic, endoscopic, cytologic, or pathologic suspicion of recurrence should prompt repeat biopsy when feasible and consideration of definitive surgical management. Potential failure criteria include documented invasive recurrence, radiologic progression, inability to safely continue surveillance, or patient preference for definitive treatment.

### 4.5 Limitations

This publication is limited by its design as a single case report. Although the patient’s response has remained durable through the most recent follow-up assessment, long-term durability data for organ preservation in UTUC are currently lacking compared with rectal cancer. Repeat PET-CT performed on 30 April 2026 demonstrated sustained complete metabolic remission without evidence of disease progression. Nevertheless, the long-term durability of organ preservation remains unknown, and late recurrence cannot be excluded. Selection bias should also be acknowledged. This patient represented a highly selected case characterized by mismatch repair deficiency, a strong preference for organ preservation, and willingness to comply with intensive surveillance. Consequently, the findings may not be generalizable to the broader population of patients with upper tract urothelial carcinoma. Therefore, these findings should be interpreted as hypothesis-generating, highlighting the need for larger prospective cohorts before widespread adoption.

## 5 Patient perspective

The patient expressed a strong preference to avoid radical nephroureterectomy and preserve renal function. He reported satisfaction with the immunotherapy-based treatment approach and accepted the need for close surveillance and frequent follow-up assessments.

## 6 Conclusions

Immunotherapy with checkpoint inhibitors may be considered a viable organ-preserving alternative to radical surgery for highly selected patients with dMMR UTUC who achieve a complete response. Supported by new prospective evidence (11), this approach offers the potential to avoid surgical morbidity without compromising oncological control. However, given the investigational nature of this strategy, strict patient selection and adherence to intensive multi-modal surveillance are essential.

## Data Availability

The original contributions presented in the study are included in the article/**Supplementary Material**. Further inquiries can be directed to the corresponding author.
