# Supplementary material for: Case Report: Kidney preservation using immune checkpoint inhibitors in Muir–Torré syndrome-associated upper tract urothelial carcinoma, with literature review
Source: Front Immunol. 2026 Jun 18;17:1784811. doi: 10.3389/fimmu.2026.1784811 (PMC13323320; doi:10.3389/fimmu.2026.1784811)
Supplement: Supplementary file 1 [file Table1.docx]

**CARE Checklist for my case report**

| **Topic** | **Item** | **Checklist Item Description** | **Reported on Section/Page** |
| --- | --- | --- | --- |
| **Title** | 1 | The diagnosis or intervention of primary focus followed by the words "case report" | **Title Page** |
| **Key Words** | 2 | 2 to 5 key words that identify diagnoses or interventions in this case report, including "case report" | **Abstract** |
| **Abstract** | 3a | Introduction: What is unique about this case and what does it add to the scientific literature? | **Abstract** |
|  | 3b | Main symptoms and/or important clinical findings | **Abstract** |
|  | 3c | The main diagnoses, therapeutic interventions, and outcomes | **Abstract** |
|  | 3d | Conclusion—What is the main "take-away" lesson(s) from this case? | **Abstract** |
| **Introduction** | 4 | One or two paragraphs summarizing why this case is unique (may include references) | **Introduction** |
| **Patient Information** | 5a | De-identified patient specific information | **Case Description** |
|  | 5b | Primary concerns and symptoms of the patient | **Case Description** |
|  | 5c | Medical, family, and psycho-social history including relevant genetic information | **Case Description** |
|  | 5d | Relevant past interventions with outcomes | **Case Description** |
| **Clinical Findings** | 6 | Describe significant physical examination (PE) and important clinical findings | **Case Description** / **Diagnostic Assessment** |
| **Timeline** | 7 | Historical and current information from this episode of care organized as a timeline | **Table 1** |
| **Diagnostic Assessment** | 8a | Diagnostic testing (such as PE, laboratory testing, imaging, surveys) | **Diagnostic Assessment** |
|  | 8b | Diagnostic challenges (such as access to testing, financial, or cultural) | **Discussion** (Surveillance Challenges) |
|  | 8c | Diagnosis (including other diagnoses considered) | **Diagnostic Assessment** |
|  | 8d | Prognosis (such as staging in oncology) where applicable | **Diagnostic Assessment** |
| **Therapeutic Intervention** | 9a | Types of therapeutic intervention (such as pharmacologic, surgical, preventive, self-care) | **Therapeutic Intervention Outcomes** |
|  | 9b | Administration of therapeutic intervention (such as dosage, strength, duration) | **Therapeutic Intervention Outcomes** |
|  | 9c | Changes in therapeutic intervention (with rationale) | **Therapeutic Intervention Outcomes** (Rationale for organ preservation) |
| **Follow-up and Outcomes** | 10a | Clinician and patient-assessed outcomes (if available) | **Therapeutic Intervention Outcomes** / **Patient Perspective** |
|  | 10b | Important follow-up diagnostic and other test results | **Therapeutic Intervention Outcomes** |
|  | 10c | Intervention adherence and tolerability (How was this assessed?) | **Therapeutic Intervention Outcomes** |
|  | 10d | Adverse and unanticipated events | **Therapeutic Intervention Outcomes** |
| **Discussion** | 11a | A scientific discussion of the strengths AND limitations associated with this case report | **Discussion** (Limitations) |
|  | 11b | Discussion of the relevant medical literature with references | **Discussion** |
|  | 11c | The scientific rationale for any conclusions (including assessment of possible causes) | **Discussion** (Biological Rationale) |
|  | 11d | The primary "take-away" lessons of this case report (without references) in a one paragraph conclusion | **Conclusions** |
| **Patient Perspective** | 12 | The patient should share their perspective in one to two paragraphs on the treatment(s) they received | **Patient Perspective** |
| **Informed Consent** | 13 | Did the patient give informed consent? Please provide if requested | **Yes (see Ethics Statement)** |
